# Altered metabolism of mothers of young children with Autism Spectrum Disorder: a case control study

**DOI:** 10.1186/s12887-020-02437-7

**Published:** 2020-12-14

**Authors:** Kathryn Hollowood-Jones, James B. Adams, Devon M. Coleman, Sivapriya Ramamoorthy, Stepan Melnyk, S. Jill James, Bryan K. Woodruff, Elena L. Pollard, Christine L. Snozek, Uwe Kruger, Joshua Chuah, Juergen Hahn

**Affiliations:** 1 Department of Biomedical Engineering, Rensselaer Polytechnic Institute, 110 8th St, Troy, NY 12180 USA; 2 School for Engineering of Matter, Transport and Energy, Arizona State University, P.O. Box 879309, ECG 302, 501 E Tyler Mall, Tempe, AZ 85287-9309 USA; 3 Metabolon, 617 Davis Drive, Suite 100, Morrisville, NC 27560 USA; 4 University of Arkansas for Medical Sciences, 4301 W Markham St, Little Rock, AR 72205 USA; 5 Mayo Clinic, 13400 E. Sea Blvd, Scottsdale, AZ 85259 USA

**Keywords:** Autism, Metabolic profile, Fisher discriminant analysis, Logistic regression, Metabolomics, Mothers

## Abstract

**Background:**

Previous research studies have demonstrated abnormalities in the metabolism of mothers of young children with autism.

**Methods:**

Metabolic analysis was performed on blood samples from 30 mothers of young children with Autism Spectrum Disorder (ASD-M) and from 29 mothers of young typically-developing children (TD-M). Targeted metabolic analysis focusing on the folate one-carbon metabolism (FOCM) and the transsulfuration pathway (TS) as well as broad metabolic analysis were performed. Statistical analysis of the data involved both univariate and multivariate statistical methods.

**Results:**

Univariate analysis revealed significant differences in 5 metabolites from the folate one-carbon metabolism and the transsulfuration pathway and differences in an additional 48 metabolites identified by broad metabolic analysis, including lower levels of many carnitine-conjugated molecules.

Multivariate analysis with leave-one-out cross-validation allowed classification of samples as belonging to one of the two groups of mothers with 93% sensitivity and 97% specificity with five metabolites. Furthermore, each of these five metabolites correlated with 8–15 other metabolites indicating that there are five clusters of correlated metabolites. In fact, all but 5 of the 50 metabolites with the highest area under the receiver operating characteristic curve were associated with the five identified groups. Many of the abnormalities appear linked to low levels of folate, vitamin B12, and carnitine-conjugated molecules.

**Conclusions:**

Mothers of children with ASD have many significantly different metabolite levels compared to mothers of typically developing children at 2–5 years after birth.

## Background

Autism spectrum disorder (ASD) involves a combination of abnormal social communication, stereotyped behaviors, and restricted interests [1]. ASD is assumed to be caused by complex interactions between genetic and environmental factors, both of which can affect metabolism. Previous studies have revealed significant abnormalities in the folate-one carbon metabolism and the transsulfuration pathways of children with ASD [2–5] and their mothers [6–8], resulting in decreased methylation capability, decreased glutathione levels, and increased oxidative stress. Furthermore, the presence of mutations in the *MTHFR* gene (A1298C and C667T) was found to be associated with increased risk of ASD [9]. The *MTHFR* gene makes an enzyme, Methylenetetrahydrofolate reductase, which converts 5,10-methylenetetrahydrofolate, a form of folate, to 5-methyltetrahydrofolate, a different form of folate. This latter form of folate is crucial in the conversion of homocysteine to methionine [10]. Additionally, levels of prenatal vitamins taken during pregnancy that include B12 and folate are associated with a decreased ASD risk [11], suggesting an association of metabolite levels of the folate one-carbon metabolism (FOCM) and the transsulfuration (TS) pathways with ASD. Studies have also found that too much folic acid supplementation can potentially lead to an increased risk of ASD [11, 12]. One of these studies suggests that folic acid and B12 supplements are associated with ASD risk by a u-shaped curve, showing that too little and too much folic acid or B12 supplementation can both lead to an increased risk of ASD [11]. Other studies found that maternal gene variants in the one-carbon metabolism pathway were associated with increased ASD risk when there was no or only low levels of periconceptional prenatal vitamin intake [13, 14].

Additional metabolic differences may also be present in mothers of children with ASD, but there has been relatively little investigation of their metabolic state. A more comprehensive understanding of metabolites and metabolic pathways of mothers of children with ASD may lead to a better understanding of the etiology of ASD and provide some insights for evaluating pre-conception risk and/or risk during pregnancy. For example, currently, the general risk of having a child with ASD in the US is approximately 1.9% [15], however, the recurrence risk increases to approximately 19% if the mother already has a child diagnosed with ASD [16].

This paper focuses on analyzing the metabolic profile of mothers of young children with ASD and mothers of typically developing children, 2–5 years after birth. Measurements were conducted with whole blood to provide information on both intra-cellular and extra-cellular metabolism. This study was limited to women who were not taking folate, B12, or multi-vitamin/ mineral supplements during the 2 months prior to sample collection, in order to minimize the effect of supplements on metabolism. The study includes assessments of many different aspects of metabolism, including analysis of amino acids, peptides, carbohydrates, lipids, nucleotides, Kreb’s cycle, vitamins/co-factors, and xenobiotics. This work is part of a larger study, the ASU-Mayo Pilot Study of Young Children with ASD and their Mothers (AMPSYCAM). Although it would be ideal to have biological samples obtained during conception, pregnancy, lactation, and infancy, this would represent a significant hurdle for study design. Instead, this pilot study focuses on 2–5 years after birth to provide preliminary insight into metabolic differences that currently exist. Results from this study provide the motivation for larger future studies to validate the findings and potentially to expand the time horizon to include the time during conception/pregnancy/lactation.

## Methods

### Study design and sample collection and analysis

#### IRB approval and consent

This study was approved by the IRB of Mayo Clinic-Arizona and the IRB of Arizona State University. All parents signed informed consent forms after the study was explained to them.

#### Advertising

The study advertisement was emailed to several thousand ASD families on the email lists of the ASU Autism/Asperger’s Research Program and the Zoowalk for Autism Research. Other local autism groups such as the Autism Society of Greater Phoenix also helped advertise the study. Finally, participants were invited to share the study advertisement with their network of friends.

#### Participants

The inclusion criteria were:

1. Mother of a child 2–5 years of age
2. Child has ASD or has typically development (TD) including both neurological and physical development
3. Child ASD diagnosis verified by the Autism Diagnostic Interview-Revised [17]

The exclusion criteria were:

1. Mother currently taking a vitamin/mineral supplement containing folic acid and/or vitamin B12
2. Mother currently taking or had taken any vitamin supplements within the past 2 months
3. Mother pregnant or planning to become pregnant in the next 6 months

The recruitment period ran from August 2016 until July 2017. Thirty mothers who have a child with ASD (ASD-M) and twenty-nine mothers who have TD children (TD-M) were recruited for this study. Originally, there were three additional ASD-M participants. However, two of the mothers were disqualified because their child did not meet the ADI-R criteria and one was disqualified because the child did not have an official ASD diagnosis by a psychiatrist, licensed psychologist, or developmental pediatrician. The mothers were age-matched by group. Enrollment was done on a rolling basis, the control group was recruited so that the two groups had a similar average age and all came from the greater Phoenix, Arizona area. All mothers in the ASD-M group had a child previously diagnosed with ASD and the diagnoses were confirmed using the ADI-R. The ADI-R is a 2-h structured parent interview and is one of the primary tools used for clinical and research diagnosis of ASD [17]. All the ADI-R interviews were conducted by Elena L. Pollard, who is a certified rater on the ADI-R and has conducted over 300 ADI-R evaluations.

#### Diet and medical history

An estimate of dietary intake during the previous week was obtained using Block Brief 2000 Food Frequency Questionnaire (Adult version), from Nutrition Quest (www.nutritionquest.com). Medical histories and current medical symptoms were collected from the mothers using a self-survey. The symptoms were collected as there is little research on the health of the mothers of children with ASD. In these surveys, pesticide exposure was defined as any pesticides used in their home during pregnancy. Furthermore, the prenatal supplement usage was recorded as whether any prenatal supplements were used, however, the specific type of prenatal supplement was not recorded. These variables are included in order to address potential cofactors in the metabolic analysis.

#### Biological sample collection

Urine collections and blood draws were conducted over a 12-month period from September 2016 to August 2017 for both groups. Most participants did their blood draws in fall/winter, with a few in spring or summer for both groups. Fasting whole blood samples were collected in the morning at the Mayo Clinic and all urine collections were first-morning. Samples were stored at -80 °C freezers at Mayo and ASU until all samples were collected, and then all samples were sent together to Metabolon for testing. The amount of time the samples were frozen ranged from 1 to 12 months with an average of 8 months.

#### Laboratory tests

Laboratory measurements were conducted by Mayo Clinic, the Metabolic and Oxidative Stress Laboratory at the University of Arkansas for Medical Sciences, and Metabolon Inc. as described below.

##### Mayo Clinic

Mayo Clinic laboratories measured levels of vitamin B12, folate, methylmalonic acid, homocysteine, isoprostane, vitamin D, vitamin E, hCG, and MTHFR variants as described below.

Vitamin B12 (cyanocobalamin) was measured quantitatively with a Beckman Coulter Access competitive binding immunoenzymatic assay. Briefly, serum is treated with alkaline potassium cyanide and dithiothreitol to denature binding proteins and convert all forms of vitamin B12 to cyanocobalamin. Cyanocobalamin from the serum competes against particle-bound anti-intrinsic factor antibody for binding to intrinsic factor – alkaline phosphatase conjugate. After washing, alkaline phosphatase activity on a chemiluminescent substrate is measured and compared against a multi-point calibration curve of known cyanocobalamin concentrations.

Folate (vitamin B9) was measured quantitatively with a Beckman Coulter Access competitive binding receptor assay. Briefly, serum folate competes against a folic acid – alkaline phosphatase conjugate for binding to solid phase-bound folate binding protein. After washing, alkaline phosphatase activity on a chemiluminescent substrate is measured and compared against a multi-point calibration curve of known folate concentrations. The Folate assay is designed to have equal affinities for Pteroylglutamic acid (Folic acid) and 5-Methyltetrahydrofolic acid (Methyl-THF), so the result is a measure of both.

Methylmalonic acid (MMA) was measured quantitatively by liquid chromatography tandem mass spectrometry (LC-MS/MS). Briefly, serum is mixed with d3-methylmalonic acid as an internal standard, isolated by solid phase extraction, separated on a C18 column, and analyzed in negative ion mode. Chromatographic conditions and mass transitions were chosen to carefully distinguish methylmalonic acid from succinic acid.

Homocysteine was measured quantitatively by LC-MS/MS. Serum is spiked with d8-homocystine as an internal standard, reduced to break disulfide bonds, and deproteinized with formic acid and trifluoroacetic acid in acetonitrile. Measurement of total homocysteine and d4-homocysteine (reduced from d8-homocystine) is performed in positive ion mode with electrospray ionization.

Urine F2-Isoprostane (8-isoprostane) was measured quantitatively by LC-MS/MS after separation from prostaglandin F2 alpha. Urine is spiked with deuterated F2-isoprostane and deuterated prostaglandin F2 alpha, then positive pressure filtered. A mixed mode anion exchange turbulent flow column is used to clean up samples which are then separated on a C8 column and analyzed in negative ion mode.

Vitamin D (25-hydroxyvitamin D2 and D3) was measured quantitatively by LC-MS/MS. D6–25-hydroxyvitamin D3 is added to serum as an internal standard before protein precipitation with acetonitrile. Online turbulent flow chromatography is used to further clean up the samples prior to separation on a C18 column and analysis in positive ion mode. The D2 and D3 forms are measured separately; results are reported as D2, D3, and the sum.

Vitamin E was measured quantitatively by LC-MS/MS. D6-alpha-tocopherol internal standard is added to serum, and proteins are precipitated with acetonitrile. The supernatant is subjected to online turbulent flow for sample cleanup, separated on a C18 column, and analyzed in positive ion mode.

Serum ferritin was measured quantitatively with a Beckman Coulter Access two-site immunoenzymatic (sandwich) assay. Serum ferritin binds mouse anti-ferritin that is immobilized on paramagnetic particles; ferritin is also bound by a goat anti-ferritin – alkaline phosphatase conjugate. After washing, alkaline phosphatase activity on a chemiluminescent substrate is measured and compared against a multi-point calibration curve of known ferritin concentrations.

MTHFR mutation analysis was performed for the A1298C and C677T variants using Hologic Invader assays. DNA was isolated from whole blood and amplified in the presence of probes for both wildtype and variant sequences. Hybridization of sequence-specific probes to genomic DNA leads to enzymatic cleavage of the probe, releasing an oligonucleotide that binds to a fluorescently labeled cassette. This second hybridization results in generation of a fluorescent signal that is specific to the wildtype or variant allele. The MTHFR gene mutations are measured as categorical variables that indicate whether a sample has the mutation.

*The Metabolic and Oxidative Stress Laboratory (MOSL)* located at Arkansas Children’s Research Institute performed the measurements described below.

##### Sample preparation for measurement of plasma methylation and oxidative stress metabolites

For concentration determination of total thiols (homocysteine, cysteine, cysteinyl-glycine, glutamyl-cysteine, and glutathione), the disulfide bonds were reduced and protein-bond thiols were released by the addition of 50 μl freshly prepared 1.43 M sodium borohydride solution containing 1.5 μM EDTA, 66 mM NaOH and 10 μl n-amyl alcohol and added to 200 μl of plasma. After gentle mixing, the solution was incubated at + 4 °C for 30 min with gentle shaking. To precipitate proteins, 250 μl ice cold 10% meta-phosphoric acid was added and the sample was incubated for 20 min on ice. After centrifugation at 18,000 g for 15 min at 4 °C, the supernatant was filtered through a 0.2 μm nylon filter and a 20 μl aliquot was injected into the high-performance liquid chromatography (HPLC) system.

For determination of free thiols and methylation metabolites, proteins were precipitated by the addition of 250 μl ice cold 10% meta-phosphoric acid and the sample was incubated for 10 min on ice. Following centrifugation at 18,000 g for 15 min at + 4 °C, the supernatant was filtered through a 0.2 μm nylon and a 20 μl aliquot was injected into the HPLC system.

##### HPLC with Coulometric electrochemical detection

The methodological details for metabolite elution and electrochemical detection have been described previously [18, 19] The analyses were accomplished using HPLC with a Shimadzu solvent delivery system (ESA model 580) and a reverse phase C_18_ column (5 μm; 4.6 × 150 mm, MCM, Inc., Tokyo, Japan) obtained from ESA, Inc. (Chelmsford, MA). A 20 μl aliquot of plasma extract was directly injected onto the column using Beckman autosampler (model 507E). All plasma metabolites were quantified using a model 5200A Coulochem II electrochemical detector (ESA, Inc., Chelmsford, MA) equipped with a dual analytical cell (model 5010) and a guard cell (model 5020). The concentrations of plasma metabolites were calculated from peak areas and standard calibration curves using HPLC software.

MOSL calculated metabolite ratios that include SAM/SAH, fGSH/GSSG, tGSH/GSSG, fCysteine/fCystine, and the percent oxidized glutathione.

##### Metabolon Inc

Metabolon Inc. conducted measurements of 595 metabolites in whole blood samples in a manner similar to a previous study [20]. Briefly, individual samples were subjected to methanol extraction then split into aliquots for analysis by ultrahigh performance liquid chromatography/mass spectrometry (UHPLC/MS). The global biochemical profiling analysis comprised of four unique arms consisting of reverse phase chromatography positive ionization methods optimized for hydrophilic compounds (LC/MS Pos Polar) and hydrophobic compounds (LC/MS Pos Lipid), reverse phase chromatography with negative ionization conditions (LC/MS Neg), as well as a hydrophilic interaction liquid chromatography (HILIC) method coupled to negative (LC/MS Polar) [21]. All of the methods alternated between full scan MS and data dependent MSn scans. The scan range varied slightly between methods but generally covered 70–1000 m/z.

Metabolites were identified by automated comparison of the ion features in the experimental samples to a reference library of chemical standard entries that included retention time, molecular weight (m/z), preferred adducts, and in-source fragments as well as associated MS spectra and curated by visual inspection for quality control using software developed at Metabolon. Identification of known chemical entities was based on comparison to metabolomic library entries of purified standards [22]. Metabolites that were not officially confirmed with a standard are marked throughout the paper with a *. Measurements that were below the detection limit were replaced with the next lowest measurement divided by the square root of two.

### Statistical analysis

#### Univariate analysis

To conduct a univariate analysis, a test was performed for whether the population means or medians between the ASD-M group and the TD-M group are equal against the alternative hypothesis that they are not. To determine which testing method to use, the Anderson-Darling test [23] was applied to each sample. If the recorded samples of a particular metabolite or ratio were drawn from two normal distributions an F-test was subsequently performed to determine whether the population variances of both distributions were identical. If at least one of the two samples of a particular metabolite or ratio was not drawn from a normal distribution, the two-sample Kolmogorov-Smirnov test [24] was applied to examine whether the two samples were drawn from unknown distributions that had the same shape. This pre-analysis yielded four distinct scenarios for a particular metabolite or ratio: (i) both samples were drawn from normal distributions that had identical population variances, (ii) both samples were drawn from a normal distribution with unequal population variances, (iii) both samples were drawn from two unknown distributions that had the same shape and (iv) both samples were drawn from distinctively different distributions. For scenarios (i), (ii), (iii) and (iv) the standard Student t-test (t=), the Welch test (t ≠) [25], the Mann-Whitney U test (MW) [26] and the Welch t-test (t ≠ †) were applied, respectively, for a significance of *α* = 0.05. If a *p*-value is less than *α*, the null hypothesis is rejected. Conversely, for a p-value above or equal to *α*, the null hypothesis cannot be rejected.

Some of the data analyzed below is categorical. In order to analyze these data, the Chi-square test (*χ*^2^) was used for independence. This tests if categorical variables are independent [27]. If this is so, the next step is to determine if the recorded categorical variables are dependent on whether the mother has previously had a child with ASD.

In order to determine the robustness of the hypothesis tests, the false discovery rates (FDR) for each metabolite were also calculated [28]. This was done by calculating the *p*-values for various combinations of mothers and calculating the fraction of p-values that were considered significant (≤ 0.05) over the total number of p-values. These combinations included leaving one mother out at a time, every combination leaving two mothers out at a time, and every combination leaving three mothers out at a time. This produced 1770 p-values for each metabolite from which the FDR was computed.

The area under the curve (AUC) of the receiver operating characteristic (ROC) curve was also calculated for each metabolite. The ROC curve is a plot of false positive rate (FPR) vs. the true positive rate (TPR). The higher the area under the curve is, the better the measurements are at classifying between the two groups of mothers [29].

A test was considered significant if the p-value was less than or equal to 0.05 and the FDR value was less than or equal to 0.1.

#### Multivariate analysis

While the univariate analyses focused on testing for equal population means or medians of individual metabolites/ratios, this does not answer the question of how important the differences in mean or median are to separate the two groups of mothers. In order to examine the extent of the differences within the recorded observations of the two groups of mothers, Fisher Discriminant Analysis (FDA) was applied [30]. This technique defines a projection direction in the data space such that the squared difference between the centers of the projected observations of both groups over the variances of the projected observations is a maximum. The objective function, *J*, to compute the projection direction is as follows:

1 $$ J=\frac{{\left({\overline{t}}_1-{\overline{t}}_2\right)}^2}{s_1^2+{s}_2^2} $$

Here, ${\overline{t}}_1=\frac{1}{n_1}{\sum}_{i=1}^{n_1}{t}_{1,i}$ and ${\overline{t}}_2=\frac{1}{n_2}{\sum}_{i=1}^{n_2}{t}_{2,i}$ are the orthogonally projected means of both groups onto the direction vector and the sample variances of the projected data points are ${s}_1^2=\frac{1}{n_1-1}{\sum}_{i=1}^{n_1}{\left({t}_{1,i}-{\overline{t}}_1\right)}^2$ and ${s}_2^2=\frac{1}{n_2-1}{\sum}_{i=1}^{n_2}{\left({t}_{2,i}-{\overline{t}}_2\right)}^2$. The orthogonal projection of *i-*th observation from the second sample, ***x***_2, *i*_, is ${t}_{2,i}={\boldsymbol{x}}_{2,i}^T\boldsymbol{p}$, where ***p*** is the unit-length direction vector. Note that the projection coordinate, *t*_2, *i*_, is often referred to as a score. Essentially, FDA produces a projection direction which represents a tradeoff between optimally separating the two groups of mothers and minimizing the spread of the projected data within each group. FDA is used to develop a multivariate model that can be used to classify between the two groups of mothers.

FDA works well with data consisting of real numbers. However, some of the data were discrete in nature such as the information about MTHFR gene mutation. For classification tasks including both continuous and discrete data, logistic regression was used. Logistic regression is similar to linear regression, but the output is a variable that can assume two or more discrete values, i.e. a binomial or multinomial variable. The prediction of a logistic regression model is the probability that a sample belongs to either the ASD-M group or the TD-M group. The group that produces the highest probability is considered the group that the model classified the sample as belonging to [31, 32].

The multivariate analysis made use of both FDA and logistic regression. The data was split into multiple subsets for analysis. These subsets include: (i) the 20 measurements from the FOCM/TS pathways, (ii) the same 20 measurements plus additional nutritional information, (iii) the 20 FOCM/TS metabolites with the additional nutritional information and the MTHFR gene information, and (iv) the 20 FOCM/TS metabolites, the additional nutritional information, the MTHFR gene information and a select number of significant metabolites from the broad metabolomics analysis. The additional nutritional markers included B12, Folate, Ferritin, Methylmalonic acid (MMA), and Vitamin E. The 50 metabolites from the Metabolon dataset included in the analysis produced were selected based upon the 50 highest AUC values from the corresponding ROC curves. These steps reduced the total number of metabolites from 621 to 76 for case iv. All combinations of two through ten variables were analyzed in each subset. FDA was used for subsets i and ii and logistic regression was used for subsets iii and iv. The reason for using two different methods is that FDA was used to ensure consistency in the methodology with prior work [8] while logistics regression was needed for subsets iii and iv because they contained the MTHFR gene information which are binary variables. The reason for analyzing a reduced set of variables for each of the four cases, instead of just investigating the full variable set, is to alleviate some of the concerns related to overfitting of the classification models.

Furthermore, a leave-one-out cross-validation procedure (LOOCV) [32] was used to independently assess classification accuracy. LOOCV removes the first observation, or participant’s data, determining a model using (Eq. 1) based on the *n* − 1 observations, and then applying this model to the first observation which was left out. This application determines whether this observation is correctly/incorrectly classified as belonging to the ASD-M or TD-M group. Then, the second observation is left out, whilst the first observation is included for determining a second model using (Eq. 1). The second model is then used to decide whether the second observation is correctly classified or misclassified. This procedure is repeated until each observation is left out once allowing the calculation of the overall rate of correctly classified and misclassified observations. To determine whether an observation is correctly or incorrectly classified, the samples describing the ASD-M group were defined as positives and the corresponding samples of the TD-M cohort as negatives. The decision boundary to assign the label “ASD-M” or “TD-M” to a data point was based on a kernel density estimation of the scores (projection coordinates) computed by the FDA model from the positives (ASD-M group). More precisely, the decision boundary is determined for a chosen confidence level (one-sided) such that a score that is less than or equal to this boundary is labeled an ASD-M subject and a score that is larger than this threshold is labeled as a TD-M subject. The confidence level is chosen to reduce the difference between the type I and type II errors.

## Results

### Univariate analysis

#### Participants

The medical histories and characteristics of the participants are shown in Tables 1, 2 and 3. The hypothesis testing shown in the tables was done using either the Chi-squared test or the Student’s t-test. Each table lists n.s. (not significant) for the *p*-value or FDR when the result was greater than 0.05 for the p-value or 0.1 for FDR, indicating that the measurement showed no statistically significant differences between the two groups. All ASD-M participants enrolled in this study had a child that met full criteria for ASD based on ADI-R scores. Table 1 lists basic characteristics and medical histories of the mothers. Information on the children can be found in the supplemental section (Table S-1) as well as information about the mother’s pregnancies (Table S-2).

**Table 1:** Characteristics and medical histories of participants. The means are shown with the standard deviations in parentheses

	ASD (***n*** = 30)	TD (***n*** = 29)	***p-***Value of t-test (T) or Chi-Squared (C)
**Maternal age (years)**	35.4 (5.2)	35.2 (5.8)	n.s. (T)
**Maternal BMI**	26.46 (5.10)	25.50 (4.65)	n.s. (T)
***Maternal Ethnicity***^a^			
**Hispanic or Latino**	22%	28%	n.s. (C)
**White**	86%	93%	n.s. (C)
**Black or African American**	0%	3%	n.s. (C)
**Asian**	4%	0%	n.s. (C)
**American Indian or Alaska Native**	0%	0%	N/A
**Native Hawaiian or Other Pacific Islander**	0%	0%	N/A
**Do not wish to provide ethnic info**	7%	0%	n.s. (C)

*A measurement was considered significant if the p-value was less than or equal to 0.05 and the FDR is less than or equal to 0.1. Standard deviations were not listed for categorical variables, marked by the usage of the Chi-Squared test (C) instead of the t-test (T)*

^a^*In reference to the ethnicity information, there were 27 respondents from the ASD-M group for the Hispanic or Latino question and 28 respondents from the ASD-M group for the rest. The ethnicity was answered by the entire TD-M group*

**Table 2:** Current maternal medication use at 2–5 years after pregnancy. The percentage of participants that used these medications is listed with the actual number in parentheses

	ASD (***n =*** 30)	TD (***n =*** 29)	Chi-Squared	FDR
**Use of Any Medications**	43% (13)	30% (9)	n.s.	
Psychiatric	17% (5)	7% (2)	n.s.	
Allergy	10% (3)	14% (4)	n.s.	
Birth control	33% (10)	10% (3)	0.03	n.s.
Inhaler	0% (0)	3% (1)	n.s.	
Blood pressure	7% (2)	0% (0)	n.s.	
Thyroid	10% (3)	7% (2)	n.s.	
GI (Gastrointestinal)	0% (0)	3% (1)	n.s.	
Pain	0% (0)	3% (1)	n.s.	
Epi-pen as needed	3% (1)	0% (0)	n.s.	
Use of Nutritional Supplements*	7% (2)	7% (2)	n.s.	

*None of the nutritional supplements used contained folate or vitamin B12*

**Table 3:** Current mental and physical symptoms of mothers. The severity scale was 0 = none, 1 = mild, 2 = moderate, 3 = severe. The mean is listed with the standard deviation in parentheses

	ASD (***n =*** 30)	TD (***n =*** 29)	Ratio of ASD/TD	t-test	FDR
**Fatigue**	1.15 (1.0)	0.79 (0.8)	1.46	n.s.	
**Fibromyalgia**	0.11 (0.6)	0 (0)		n.s.	
**Depression**	0.67 (0.8)	0.48 (0.7)	1.38	n.s.	
**Irritability**	0.78 (0.9)	0.71 (0.8)	1.09	n.s.	
**Anxiety**	1.11 (0.9)	0.75 (1.0)	1.48	0.08	n.s.
**Cognition**	0.56 (1.0)	0.32 (0.6)	1.73	n.s.	
**Attention**	0.85 (1.0)	0.32 (0.55)	2.65	0.056	n.s.
**Sensory Sensitivity**	0.44 (0.7)	0.18 (0.4)	2.49	n.s.	
**Stool/GI Problems**	0.44 (0.8)	0.71 (1.0)	0.62	n.s.	
**Sleep Problems**	0.74 (0.9)	0.46 (0.74)	1.60	n.s.	
**Headaches**	0.56 (0.8)	0.64 (0.7)	0.86	n.s.	
**Chemical Sensitivity**	0.19 (0.5)	0.14 (0.5)	1.30	n.s.	
**Allergies**	0.78 (1.0)	0.71 (0.7)	1.09	n.s.	
**Average**	0.60 (0.5)	0.44 (0.4)	1.37	n.s.	

*Other symptoms not listed were also reported. For the ASD-M group, multiple sclerosis, very sensitive to alcohol, and frequent boils were reported. For the TD-M group, nausea/pain from ovarian cyst, chronic pain, sensitive to loud noises, and touch aversive were reported. The symptoms were considered significantly different between the two groups if the p-value was less than or equal to 0.05 and the FDR was less than or equal to 0.1. GI in this case stands for gastrointestinal. The ratio of ASD/TD values refers to the ratio of the means of the data for ASD cases and TD cases*

The average age of mothers in the ASD-M group and TD-Group were similar (35.4 years and 34.9 years, respectively), since they were matched for maternal age. The average ages of the children were slightly older for the ASD group (4.71 vs. 3.87 years), as any age between 2 and 5 years was allowed, and the ASD group was skewed towards the end of that range since it takes time for children with ASD to be diagnosed and to have been contacted for this study (Table S-2).

More information on the mothers can be found in Tables 2 and 3. Table 2 lists the medications that were taken by the mothers at the time of the study and Table 3 lists mental and physical symptoms of the mothers. The *p*-values and FDR results in Tables 2 and 3 show that there were no significant differences in the medication use listed and the symptoms experienced between the two groups of mothers during the study period.

#### FOCM/TS metabolites

The univariate results for the FOCM/TS metabolites are shown in Table 4. Levels of vitamin B12 and the SAM/SAH ratios are significantly lower in the ASD-M group compared to the TD-M group, (p ≤ 0.05, FDR ≤ 0.1). Also, levels of Glu-Cys, fCysteine, and fCystine are significantly higher in the ASD-M group compared to the TD-M group (p ≤ 0.05, FDR ≤ 0.1).

**Table 4:** Univariate results for FOCM/TS metabolites and vitamin E, folate, ferritin, B12, MMA, and MTHFR status

Metabolite	Test	ASD-M (mean ***±*** std) ***N*** = 30	TD-M (mean ***±*** std) ***N*** = 29	***p-***Values	FDR	AUC	Ratio (ASD-M/TD-M)
B12 Δ	MW	355 ± 196	473 ± 173	2.40E-03	0.00	0.73	0.75
fCysteine Δ	t=	23.8 ± 1.88	22.6 ± 1.94	0.01	0.00	0.70	1.06
Glu-Cys Δ	t=	1.89 ± 0.22	1.72 ± 0.24	0.01	0.00	0.69	1.10
SAM/SAH Δ	t=	1.94 ± 0.25	2.09 ± 0.20	0.01	0.00	0.67	0.93
fCystine Δ	t=	24.1 ± 2.78	22.4 ± 2.43	0.02	0.01	0.67	1.07
tCysteine	t=	248 ± 23.1	234 ± 28.3	0.04	0.40	0.64	1.06
tGSH	t=	6.23 ± 0.98	5.85 ± 1.07	0.15	1.00	0.63	1.07
SAM	t=	47.2 ± 5.37	49.3 ± 5.76	0.15	1.00	0.62	0.96
Methionine	t=	19.9 ± 2.56	20.8 ± 2.98	0.19	1.00	0.61	0.95
MTHFR mut. (A1298C)	*χ*^2^			0.15	1.00	0.60	
tGSH/GSSG	t=	29.5 ± 6.21	27.3 ± 6.06	0.18	1.00	0.60	1.08
Folate	MW	17.6 ± 6.19	21.1 ± 9.17	0.20	1.00	0.60	0.83
Homocysteine	MW	8.63 ± 0.98	8.27 ± 1.18	0.21	1.00	0.60	1.04
tGSH/GSSG	t=	29.5 ± 6.21	27.3 ± 6.06	0.18	1.00	0.60	1.08
SAH	t=	24.5 ± 2.66	23.6 ± 2.04	0.15	1.00	0.59	1.04
Vitamin D3‡	t=	27.1 ± 9.10	24.9 ± 6.08	0.27	1.00	0.57	1.09
Ferritin	MW	35.1 ± 31.0	29.5 ± 26.1	0.36	1.00	0.57	1.19
Cys-Gly	t=	38.7 ± 5.06	37.6 ± 6.38	0.46	1.00	0.57	1.03
Adenosine	t=	0.22 ± 0.03	0.21 ± 0.03	0.28	1.00	0.55	1.04
fGSH/GSSG	MW	8.73 ± 2.08	8.81 ± 1.84	0.49	1.00	0.55	0.99
% Oxidized GSH	MW	0.19 ± 0.03	0.19 ± 0.04	0.49	1.00	0.55	1.01
Chlorotyrosine	t=	26.8 ± 4.27	27.6 ± 4.23	0.51	1.00	0.55	0.97
Nitrotyrosine	t ≠	32.9 ± 6.28	33.7 ± 4.67	0.59	1.00	0.55	0.98
fGSH	t=	1.85 ± 0.32	1.89 ± 0.35	0.62	1.00	0.55	0.98
fCystine/fCysteine	t=	1.01 ± 0.11	1.00 ± 0.10	0.59	1.00	0.54	1.02
Vitamin E	t=	9.23 ± 2.53	9.82 ± 3.22	0.75	1.00	0.54	0.94
Isoprostane (U)‡	MW	0.15 ± 0.10	0.18 ± 0.14	0.70	1.00	0.53	0.83
MTHFR mut. (C677T)	*χ*^2^			0.90	1.00	0.53	
GSSG	t=	0.22 ± 0.04	0.22 ± 0.03	0.93	1.00	0.52	1.00
MMA	MW	0.15 ± 0.06	0.15 ± 0.08	0.64	1.00	0.51	0.96

*The measurements are ordered by decreasing AUC. Statistically significant metabolites with p-value ≤ 0.05 and FDR ≤ 0.1 are marked with* Δ *and ‡ indicates measurements that were left out of the classification procedure as the measurements were not collected from all mothers. Specifically, these were vitamin D with 28 mothers in ASD-M and 28 TD-M mothers and Isoprostane with 28 participants that were ASD-M and 25 mothers in TD-M. The abbreviated metabolites are defined as follows: S-Adenosylhomocysteine (SAH), S-Adenosylmethionine (SAM), glutathione (GSH), oxidized glutathione (GSSG), and Methylmalonic acid (MMA). The metabolite ratios include SAM/SAH, tGSH/GSSG, fGSH/GSSG, and percent oxidized GSH. The “f” and the “t” refer to free and total, respectively. The possible hypothesis tests were Mann-Whitney U (MW), Student’s t-test (t=), Chi-Squared test (χ*^2^*), and Welch’s test (t* ≠*)*

#### Global metabolic profile- Metabolon

622 metabolites were measured in whole blood. The univariate analysis for the 50 metabolites from broad metabolomics with the highest AUC values are shown in Table 5. They are ordered starting with those with the highest AUC. Note that these are semi-quantitative measurements (no absolute values), so only the ratio of ASD-M/TD-M is shown. In almost every case the ASD-M group had lower levels of metabolites than the TD-M group, with the levels of 4-vinylphenol sulfate, NAD+, and three glycine-containing metabolites (gamma-glutamylglycine, cinnamoylglycine, propionylglycine) being especially low (ASD-M/TD-M ratio < 0.5). Four metabolites were higher in the ASD-M group (histidylglutamate, asparaginylalanine, dimethyl sulfone, and mannose). Note that dimethyl sulfone was unusually high in the ASD-M group (ASD-M/TD-M ratio = 18.7, *p* = 0.01, but the FDR was not significant), 80% of the TD-M measurements of dimethyl sulfone and 47% of the ASD-M measurements of dimethyl sulfone were below the detection limit, and the distribution of the data for it is skewed.

**Table 5:** Univariate results of the metabolites from broad metabolomics

Metabolite	Test	ASD-M (mean ± std) ***N =*** 30	TD-M (mean ***±*** std) ***N =*** 29	***p-***Value	FDR	AUC	Ratio (ASD-M/TD-M)
Fructose Δ	t ≠ †	2.08E6 ± 7.88E5	3.61E6 ± 2.07E6	6.88E-04	0.00	0.81	0.60
Histidylglutamate Δ	t ≠	1.05E5 ± 3.25E4	7.27E4 ± 2.05E4	2.67E-05	0.00	0.80	1.50
Decanoylcarnitine (C10) Δ ^b^	t ≠	3.73E6 ± 1.48E6	5.85E6 ± 2.36E6	1.55E-04	0.00	0.78	0.66
S-1-pyrroline-5-carboxylate Δ	MW	1.62E5 ± 1.06E5	2.65E5 ± 1.14E5	3.09E-04	0.00	0.77	0.63
Octanoylcarnitine (C8) Δ ^b^	MW	1.96E6 ± 7.67E5	3.00E6 ± 1.17E6	4.00E-04	0.00	0.77	0.67
4-vinylphenol sulfate Δ	t ≠ †	6.52E5 ± 6.79E5	2.20E6 ± 2.27E6	1.30E-03	0.00	0.77	0.31
Cis-4-decenoylcarnitine (C10:1) Δ ^b^	t=	2.25E6 ± 7.42E5	3.12E6 ± 1.07E6	5.82E-04	0.00	0.74	0.75
N-formylanthranilic acid Δ	t=	1.12E5 ± 5.20E4	1.67E5 ± 7.04E4	1.20E-03	0.00	0.74	0.69
N-acetylasparagine Δ	t ≠ †	3.31E5 ± 6.33E4	4.37E5 ± 1.60E5	1.90E-03	0.00	0.73	0.78
Arachidoylcarnitine (C20)^a^ Δ ^b^	MW	4.68E5 ± 2.09E5	5.80E5 ± 1.75E5	3.00E-03	0.00	0.73	0.85
N-palmitoylglycine Δ	t=	7.60E4 ± 2.51E4	9.76E4 ± 2.65E4	2.20E-03	0.00	0.72	0.81
Citrulline Δ	t=	3.36E7 ± 4.90E6	3.82E7 ± 6.37E6	2.60E-03	0.00	0.72	0.91
6-hydroxyindole sulfate Δ	t ≠	2.94E5 ± 1.56E5	5.16E5 ± 3.07E5	1.20E-03	0.00	0.72	0.59
N-palmitoylserine Δ	MW	6.90E4 ± 3.61E4	9.33E4 ± 3.78E4	4.00E-03	0.00	0.72	0.77
Myristoylcarnitine (C14) Δ ^b^	MW	2.61E6 ± 1.21E6	3.30E6 ± 1.17E6	4.10E-03	0.00	0.72	0.82
Laurylcarnitine (C12) Δ ^b^	MW	1.41E6 ± 5.33E5	1.96E6 ± 8.25E5	4.30E-03	0.00	0.72	0.74
Stearoylcarnitine (C18) Δ ^b^	MW	2.15E7 ± 8.37E6	2.61E7 ± 6.68E6	0.01	0.00	0.71	0.85
Gamma-glutamylglycine Δ	t ≠ †	2.91E4 ± 5.86E4	1.12E5 ± 1.24E5	2.20E-03	0.00	0.71	0.27
5-oxoproline Δ	t=	9.59E6 ± 1.30E6	1.05E7 ± 1.29E6	0.01	0.00	0.70	0.94
Asparaginylalanine Δ	t=	8.80E4 ± 2.68E4	6.89E4 ± 2.16E4	3.90E-03	0.00	0.70	1.32
Glutamine Δ	t=	4.11E8 ± 5.43E7	4.46E8 ± 5.38E7	0.02	0.00	0.70	0.95
Catechol sulfate Δ	MW	2.10E6 ± 1.54E6	3.22E6 ± 1.83E6	0.01	0.00	0.70	0.67
3-indoxyl sulfate Δ	t ≠	7.41E6 ± 3.56E6	1.15E7 ± 5.98E6	2.70E-03	0.00	0.70	0.67
7-methylxanthine Δ	MW	1.56E5 ± 3.18E5	2.75E5 ± 2.48E5	0.01	0.00	0.70	0.58
Phenol sulfate Δ	MW	1.22E7 ± 7.67E6	1.88E7 ± 1.21E7	0.01	0.00	0.70	0.67
Cinnamoylglycine Δ	t ≠ †	1.73E5 ± 1.59E5	3.87E5 ± 4.08E5	0.01	0.00	0.70	0.46
Alpha-ketoglutaramate^a^ Δ	t=	2.53E6 ± 1.46E6	3.59E6 ± 1.77E6	0.02	0.00	0.70	0.73
Isovalerylglycine Δ	MW	5.91E4 ± 2.88E4	7.81E4 ± 2.82E4	0.01	0.00	0.69	0.78
Propionylglycine Δ	MW	9.15E4 ± 1.19E5	1.97E5 ± 1.89E5	0.01	0.00	0.69	0.48
Docosapentaenoylcarnitine (C22:5n3)^a^ Δ ^b^	MW	1.61E5 ± 1.16E5	2.31E5 ± 1.39E5	0.01	0.00	0.69	0.72
N-acetyl-2-aminooctanoate^a^ Δ	t ≠	7.20E4 ± 4.51E4	1.38E5 ± 1.05E5	3.20E-03	0.00	0.69	0.54
S-methylglutathione Δ	t=	2.06E5 ± 5.87E4	2.47E5 ± 7.06E4	0.02	0.01	0.69	0.86
Gamma-glutamyltyrosine Δ	MW	4.25E4 ± 3.40E4	6.83E4 ± 3.29E4	0.02	0.00	0.68	0.64
Succinylcarnitine (C4-DC) Δ ^b^	t=	2.80E6 ± 8.21E5	3.31E6 ± 9.04E5	0.03	0.07	0.68	0.87
Arachidonoylcarnitine (C20:4) Δ ^b^	MW	1.10E6 ± 5.59E5	1.47E6 ± 8.12E5	0.02	0.00	0.68	0.77
Glycine Δ	t=	2.04E7 ± 4.44E6	2.42E7 ± 6.07E6	0.01	0.00	0.68	0.87
N-acetylvaline	t=	7.51E4 ± 4.01E4	9.74E4 ± 3.92E4	0.04	0.28	0.68	0.80
Lignoceroylcarnitine (C24)^a^ Δ ^b^	t=	6.12E5 ± 1.72E5	7.48E5 ± 2.61E5	0.02	0.01	0.68	0.84
Guaiacol sulfate Δ	MW	4.23E5 ± 4.12E5	5.42E5 ± 3.12E5	0.02	0.01	0.68	0.81
5-methylthioadenosine (MTA) Δ	MW	2.60E5 ± 1.28E5	3.12E5 ± 1.11E5	0.02	0.00	0.68	0.86
Proline Δ	MW	2.44E8 ± 4.76E7	2.80E8 ± 5.83E7	0.02	0.00	0.68	0.90
Pyridoxate Δ	MW	6.26E6 ± 2.78E5	8.61E5 ± 4.53E5	0.02	0.00	0.68	0.75
Palmitoylcarnitine (C16) Δ ^b^	MW	3.12E7 ± 8.25E6	3.89E7 ± 1.33E7	0.02	0.03	0.67	0.83
Eicosenoylcarnitine (C20:1)^a^ Δ ^b^	MW	1.46E6 ± 4.30E5	1.82E6 ± 7.46E5	0.02	0.05	0.67	0.83
Nicotinamide adenine dinucleotide (NAD+) Δ	t ≠ †	5.53E4 ± 5.46E4	1.39E5 ± 1.91E5	0.03	0.05	0.67	0.41
Dimethyl sulfone	MW	6.06E5 ± 2.08E6	3.36E4 ± 1.04E5	0.01	0.23	0.67	18.7
Tiglylcarnitine (C5:1-DC) Δ ^b^	MW	3.18E4 ± 3.19E4	5.19E4 ± 3.40E4	0.02	0.02	0.67	0.63
Adrenoylcarnitine (C22:4)^a b^	MW	2.67E5 ± 1.50E5	3.75E5 ± 2.38E5	0.03	0.11	0.67	0.74
3-methylxanthine	MW	2.32E5 ± 3.76E5	3.22E5 ± 3.10E5	0.03	0.13	0.67	0.74
Mannose	MW	1.49E7 ± 3.96E6	1.27E7 ± 2.19E6	0.03	0.19	0.67	1.21

*The metabolites listed here are the 50 metabolites measured by Metabolon from broad metabolomics with the highest area under the receiver operating characteristic (ROC) curve (AUC). Metabolites with p-value ≤ 0.05 and FDR ≤ 0.1 marked with* **Δ**. *The possible hypothesis tests include Welch’s test without the normality criteria being met (t* ***≠*** *†), Welch’s test (t* ***≠****), Mann-Whitney U (MW, and Student’s t-test (t=). The* ^a^ *indicates metabolites that have not been officially confirmed based on a standard, but Metabolon is confident in the metabolite’s identity. The* ^b^ *indicates carnitine-conjugated metabolites*

Hypothesis testing was also done on the entire Metabolon dataset and revealed that 48 of these metabolites had significant differences between the two groups of mothers. Three of these metabolites were not included in the top 50 used for analysis because they had lower AUC values than the metabolites included (see Table S-3). The pathways and subpathways of these metabolites can be found in the supplemental section in Table S-4.

#### Carnitine

As shown in Table 5, several carnitine-conjugated metabolites are significantly different in the two groups of mothers. These metabolites have been indicated in Table 5 with #. The ratio of ASD/TD for carnitine-conjugated metabolites was consistently low, ranging from 0.63 to 0.87, with an average of 0.77. There were 33 additional carnitine metabolites in the 600 metabolites measured by untargeted metabolomics. Of these 33, eight metabolites had ratios indicating that the levels of carnitine-conjugated molecules in the ASD-M group were significantly less than in the TD-M group, and none were significantly higher.

Additional univariate hypothesis testing on carnitine and two of its precursors (lysine and trimethyllysine) from the Metabolon dataset not included in the top 50 found that the levels of these metabolites are very similar in the ASD-M and TD-M groups (within 1%; data not shown). This suggests that the low levels of carnitine-conjugated metabolites is not due to a carnitine deficiency.

Since the levels of carnitine-conjugated molecules were lower in the ASD-M group (see Table 5), and since beef is the primary dietary source of carnitine (some can also be made by the body), hypothesis testing was performed on the beef quantity and beef frequency in the mother’s diets to see if there was a difference between the two groups of mothers. These results are shown in Table 6 below.

**Table 6:** Univariate hypothesis testing results for beef intake of mothers during pregnancy

Variable	Test	ASD-M (mean ***±*** std) ***N =*** 28	TD-M (mean ***±*** std) ***N =*** 29	***p-***Value	FDR	AUC	Ratio (ASD-M/TD-M)
Beef Frequency	MW	2.50 ± 1.43	2.24 ± 0.91	0.73	1.00	0.53	1.12
Beef Quantity	MW	1.46 ± 0.58	1.41 ± 0.50	0.83	1.00	0.51	1.41

*The beef frequency and quantity measurements were not recorded for every ASD-M participant which is why N = 28 in this case. The beef frequency is defined as the number of times beef was eaten per week and the beef quantity is defined as the serving size (compared to a standard serving)*

There was no significant difference found in the mean/median of the beef consumption frequency and quantity between the two groups. Also, the beef consumption frequency and quantity measurements did not significantly correlate with carnitine levels, except for a slight negative correlation of beef frequency and lignoceroylcarnitine (C24) (*r =* − 0.26, *p* = 0.05, unadjusted; data not shown).

### Multivariate analysis

The multivariate analysis was performed using multiple subsets of data. The subsets included the twenty metabolites from the FOCM/TS pathways (i), the FOCM/TS metabolites plus some additional nutritional information (ii), the FOCM/TS metabolites plus the additional nutritional information and the MTHFR gene information (iii), and subset iii plus fifty metabolites from the broad metabolomics analysis (iv). The first two subsets were analyzed using FDA because all of the variables were continuous, and to allow comparison with a previous study [8], and the last two subsets were analyzed using logistic regression because the variables included both continuous and binary data. Each multivariate analysis was combined with leave-one-out cross-validation in order to ensure a statistically independent evaluation of classifiers obtained. The best combinations of metabolites from each of the first three subsets had misclassification errors ranging from 20 to 27% which shows only a very modest ability to predict which group of mothers the sample came from. The highest accuracies were found when analyzing the fourth and final dataset with errors of 3%. The best combinations of fewer metabolites are included in the supplementary section (Table S-5). Combinations that contained more than 5 variables resulted in a decrease in accuracy due to overfitting of the classification model. It is important to note that many other combinations of metabolites yielded similar results as the top combination of five metabolites, but the errors were slightly higher (Table S-6). Table 7 below details the type I/type II errors using these metabolites.

**Table 7:** Results for the combinations of metabolites from all subsets (i-iv) with lowest errors

Subset	Combination	Type I Error	Type II Error
(i): FOCM/TS Metabolites	tCysteine, Glu-Cys, fCysteine, fCystine/fCystiene, Nitrotyrosine	24%	27%
(ii): FOCM/TS metabolites plus nutritional information	SAM/SAH, Glu-Cys, GSSG, fCysteine, B12	24%	27%
(iii): FOCM/TS metabolites, nutritional information, and MTHFR gene information	SAM/SAH, tCysteine, Glu-Cys, B12, MTHFR mut. (A1298C)	24%	20%
(iv): FOCM/TS metabolites, nutritional information, MTHFR gene information, and Metabolon metabolites	Glu-Cys, histidylglutamate, cinnamoylglycine, proline, adrenoylcarnitine (C22:4)^a^	3%	3%

*The* ^a^ *indicates metabolites measured by Metabolon that were not officially confirmed based on a standard, but Metabolon is confident of the Metabolite’s identity*

**Fig. 1:**
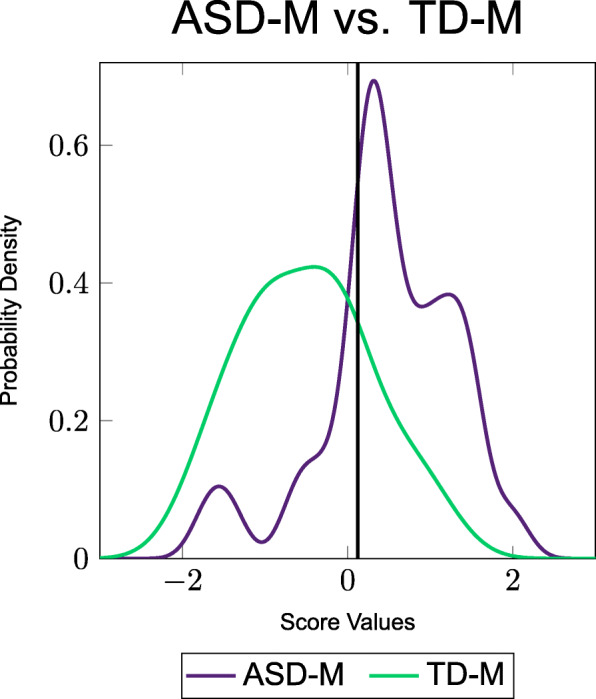
PDFs of the combination of metabolites from the FOCM/TS metabolites (i) that resulted in the respective errors shown in Table 7

**Fig. 2:**
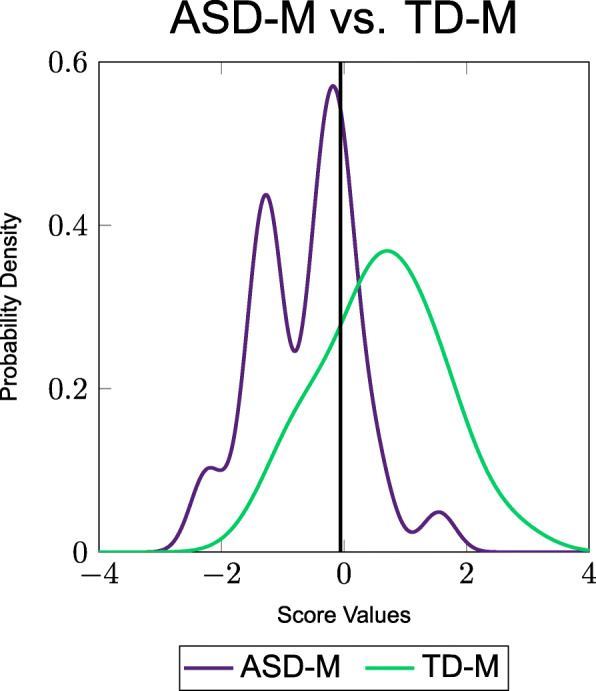
PDFs of the combination of metabolites from the FOCM/TS metabolites and additional measurements (ii) that resulted in the respective errors shown in Table 7

In order to visually demonstrate the separation between the two groups, a probability density function (PDF) was created for each of the best combinations analyzed using FDA. These PDFs are shown in Figs. 1 and 2.

**Fig. 3:**
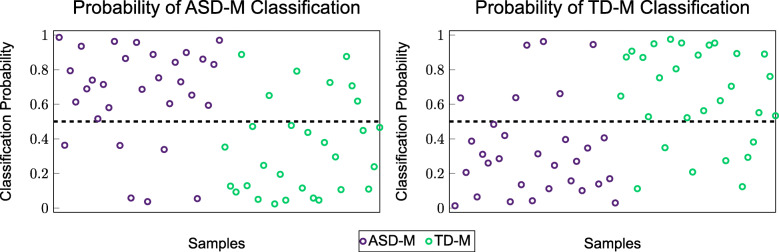
Scatter plot of the probabilities of being classified into one group or the other using a combination of variables from the FOCM/TS pathways, the additional measurements, and the MTHFR gene information (iii) that resulted in the errors listed in Table 7

In order to visually demonstrate the classification accuracy between the two groups when using the logistic regression classification model, a scatter plot was created showing the probabilities of each sample being classified as one group or another. The scatter plot representing the combination of metabolites from the FOCM/TS metabolites plus additional information and the MTHFR gene information (iii) is shown in the Fig. 3 below.

**Fig. 4:**
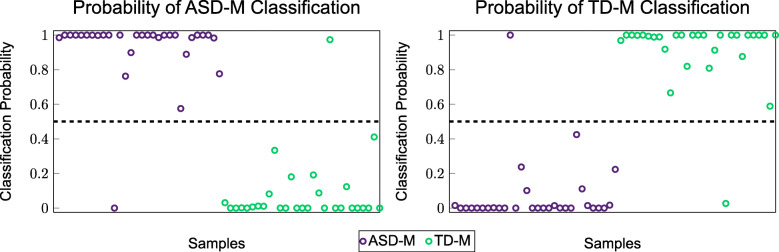
Scatter plot of the probabilities of being classified into one group or the other using a combination of variables from the FOCM/TS pathways, the additional measurements, and the top 50 metabolites from the metabolon

To further illustrate classification accuracy of the 5-metabolite model from Table 7 the probabilities that the samples would be classified by the model in each of the two groups are shown in Fig. 4. The metabolites of this 5-metabolite model consisting of Glu-Cys, histidylglutamate, cinnamoylglycine, proline, adrenoylcarnitine (C22:4)* are hereafter referred to as the “core metabolites” as these resulted in the lowest type I and type II errors.

The plots show that the ASD-M samples have a high probability of being classified as ASD-M and the TD-M samples have a high probability of being classified as TD-M. The results from this figure coupled with the low misclassification errors from Table 7 show that there are significant metabolic differences between the two groups of mothers and that these differences are sufficiently large to allow for accurate classification in the vast majority of cases.

In order to further investigate the differences between the two groups, the correlation coefficients between the 5 metabolites from the best classification model (Table 7) and the rest of the metabolites considered in the analysis for the combined set of ASD-M and TD-M samples were calculated. The metabolites that had the highest correlation coefficients with these metabolites are listed in Table 8. Furthermore, the correlations of the top 5 metabolites with one another were calculated, and, as expected, very little correlation among these five were found (see Table 9); this is not unexpected as the classification algorithms tries to identify metabolites that provide new information that can be used for classification as redundant information will not increase classification accuracy. This suggests that there are five general areas of metabolic differences in mothers of children with/without ASD involving 9 or more metabolites for each area.

**Table 8:** Correlations between the five core metabolites and the other 71 analyzed metabolites

Metabolite	Correlation Coefficient	***p-***Value
***Glu-Cys***		
tGSH	0.55	5.71E-06
tGSH/GSSG	0.35	0.01
6-hydroxyindole sulfate	−0.25	0.05
SAM/SAH	−0.26	0.04
N-formylanthranilic acid	−0.28	0.03
5-methylthioadenosine (MTA)	−0.28	0.03
Pyridoxate	−0.31	0.02
Folate	−0.38	3.40E-03
***Histidylglutamate***		
Asparaginylalanine	0.55	6.74E-06
Mannose	0.40	1.70E-03
fCystine	0.30	0.02
Succinylcarntine (C4-DC)	−0.27	0.04
Citrulline	−0.27	0.04
Fructose	−0.28	0.03
Octanoylcarnitine (C8)	−0.29	0.02
Gamma-glutamylglycine	−0.30	0.02
Isovaleryglycine	−0.32	0.01
Decanoylcarnitine (C10)	−0.33	0.01
***Cinnamoylglycine***		
N-acetyl-2-aminooctanoate^a^	0.45	4.13E-04
N-formylanthranilic acid	0.44	4.30E-04
3-indoxyl sulfate	0.35	0.01
Citrulline	0.33	0.01
6-hydroxyindole sulfate	0.32	0.01
Chlorotyrosine	0.29	0.02
Alpha-ketoglutaramate^a^	0.28	0.03
Nicotinamide adenine dinucleotide (NAD+)	0.28	0.03
Pyridoxate	0.27	0.04
Guaiacol sulfate	0.26	0.04
S-methylglutathione	0.26	0.04
Methionine	−0.29	0.02
fCysteine	−0.33	0.01
***Proline***		
S-1-pyrroline-5-carboxylate	0.59	1.22E-06
Gamma-glutamyltyrosine	0.45	3.73E-04
3-indoxyl sulfate	0.44	5.33E-04
6-hydroxyindole sulfate	0.43	6.01E-04
Phenol sulfate	0.41	1.20E-03
Glutamine	0.36	0.01
Propionylglycine	0.35	0.01
Glycine	0.35	0.01
Gamma-glutamylglycine	0.32	0.01
5-oxoproline	0.30	0.02
Alpha-ketoglutaramate^a^	0.28	0.03
Folate	0.28	0.03
N-formylanthranilic acid	0.27	0.04
Adenosine	−0.30	0.02
***Adrenoylcarnitine (C22:4)***^a^		
Arachidonoylcarnitine (C20:4)	0.93	8.00E-26
Docosapentaenoylcarnitine (C22:5n3)^a^	0.85	8.64E-18
Eicosenoylcarnitine (C20:1)^a^	0.74	2.35E-11
Palmitoylcarnitine (C16)	0.70	8.13E-10
Myristoylcarnitine (C14)	0.69	2.17E-09
Laurylcarnitine (C12)	0.49	8.88E-05
Fructose	0.41	1.20E-03
N-acetylasparagine	0.38	2.60E-03
Stearoylcarnitine (C18)	0.31	0.02
Methionine	0.26	0.04
Cys-Gly	0.26	0.05
Arachidoylcarnitine (C20)^a^	0.26	0.05
N-palmitoylserine	0.25	0.05
fCysteine/fCystine	−0.29	0.03
fCystine	−0.30	0.02

*The* ^a^ *indicates metabolites measured by Metabolon that have not been officially confirmed by a standard, but Metabolon is confident in the metabolite’s identity*

**Table 9:** Correlation coefficients between the five core metabolites model from Table 7 that provide the highest accuracy

Metabolites	Correlation Coefficient	***P-***value
Glu-Cys x Histidylglutamate	−0.01	0.92
Glu-Cys x Cinnamoylglycine	−0.06	0.63
Glu-Cys x Proline	−0.09	0.51
Glu-Cys x Adrenoylcarnitine (C22:4)^a^	0.01	0.95
Histidylglutamate x Cinnamoylglycine	−0.03	0.81
Histidylglutamate x Proline	−0.07	0.59
Histidylglutamate x Adrenoylcarnitine (C22:4)^a^	−0.06	0.65
Cinnamoylglycine x Proline	1.80E-03	0.99
Cinnamoylglycine x Adrenoylglycine (C22:4)^a^	−0.13	0.31
Proline x Adrenoylcarnitine (C22:4)^a^	0.04	0.74

*The* ^a^ *indicates metabolites measured by Metabolon that have not been officially confirmed based on a standard, but Metabolon is confident in the Metabolite’s identity*

Most of the metabolites listed in Tables 4 and 5 that were significantly different between the ASD and TD groups were found to be significantly correlated with the 5 core metabolites. However, there were 5 metabolites that were significantly different between the ASD-M and TD-M groups that did not significantly correlate with the 5 core metabolites. These five metabolites were B12, cis-4-decenoylcarnitine (C10:1), catechol sulfate, 7-methylxanthine, and tiglylcarnitine (C5:1-DC). A correlation analysis was conducted to determine if any of the 5 metabolites were correlated with one another, possibly forming a 6th group of correlated metabolites. However, none of the 5 metabolites were significantly correlated with one another. So, it appears that there are 5 primary sets of metabolites, and 5 additional metabolites that are not part of those 5 groups, which are significantly different between the ASD-M and TD-M groups.

Overall, many of the metabolites measured in this study are significantly different between the two groups of mothers, ASD-M and TD-M. The subset of metabolites that worked the best for classification was a subset of five metabolites which were each correlated with many others.

## Discussion

### Univariate analysis

The hypothesis testing done on the medical histories, current medications, and symptoms indicated that there were no significant differences between the two groups of mothers other than the age of their children. Therefore, the medical histories, current medications, and symptoms were determined to not have a large effect on the differences in metabolite measurements between the two different groups in this particular study. The *p*-value was significant for the use of birth control, but the FDR value was not. The slightly higher use of birth control in the ASD group may be due to less desire to have additional children after one child is already diagnosed with ASD. This shows that the differences that were found in the metabolites were most likely not due to the age of the mothers, their medical histories, current medications, or current symptoms. Hypothesis testing on the metabolites from the FOCM/TS pathways, additional nutritional information, and MTHFR gene information revealed that only five of these measurements have a significant difference in the mean/median between the two groups (Table 4). A meta-analysis of 12 studies [33] found that supplementation with folic acid during pregnancy results in a significantly reduced risk of ASD in the children, with some studies suggesting that folic acid supplementation during the first 2 months of pregnancy is most important. Levels of folate were not significantly lower in the ASD-M group in this study (17% lower, *p* = 0.20, n.s.), but folate levels were significantly correlated with two of the five key metabolites (Glu-Cys and proline; data not shown). Similarly, vitamin B12 levels were significantly lower in the ASD-M group, and significantly correlated with six of the top 50 metabolites, and one study [11] found that abnormal maternal levels of vitamin B12 were associated with an increased risk of ASD, although one small study [34] found no association. Vitamin B12 and folate work together in recycling of homocysteine to methionine, a key step of the FOCM/TS pathway.

Hypothesis testing was next performed on the 50 metabolites from Metabolon with the highest AUC. Forty-five of these 50 metabolites were found to have significant differences (p ≤ 0.05, FDR ≤ 0.1) between the two groups of mothers. Additionally, three other metabolites, not found among the 50 with the highest AUC, also showed statistically significant differences between the two groups (see Table S-3). This reveals that, in addition to the known abnormalities in the FOCM/TS pathway [2–8] there are also many other metabolic pathway differences between mothers of children with/without ASD. The pathways of the significantly different metabolites are listed in the supplemental section in Table S-4. The primary categories of these metabolites are amino acids, carnitines, and xenobiotics. In almost all cases these particular metabolites were significantly lower in the ASD-M group. This does not appear to be an artifact of the study, because all samples were collected identically and processed and analyzed together, and most metabolites were not significantly different between the ASD-M and TD-M groups. So, the large number of metabolites listed in Table 5 suggest that there are in fact many metabolic differences between the ASD-M and TD-M groups.

### Multivariate analysis

Multivariate analysis was performed to investigate if the metabolites measured would be able to classify a mother as either having had a child with ASD (ASD-M) or a typically-developing child (TD-M). When using just the metabolites from the FOCM/TS metabolites, a combination of five metabolites (tCysteine, Glu-Cys, fCysteine, fCystine/fCysteine, and Nitrotyrosine) appeared to have the lowest misclassification errors calculated using leave-one-out cross-validation with errors of approximately 25% These errors show that the first subset of metabolites have only modest ability to classify the two groups of mothers. It is interesting to note that the present results for the FOCM/TS analysis revealed substantially less ability to distinguish the ASD mothers than a similar study. This previous study found that metabolites from the FOCM/TS pathways could classify between pregnant mothers who have had a child with ASD and pregnant mothers who have not with an accuracy of about 90% [8]. The key difference is that the present paper analyzed FOCM/TS metabolites 2–5 years after birth, whereas the other study evaluated mothers during pregnancy; in other words, measurements during pregnancy were better predictors of ASD risk. The addition of other biomarkers (B12, folate, Ferritin, MMA, vitamin E, and MTHFR) to the dataset did not significantly improve classification with either FDA or logistic regression.

The fourth subset of metabolites included the FOCM/TS metabolites, the nutritional biomarkers, the MTHFR gene information, and 50 metabolites from the 600 metabolites measured by Metabolon. Using this larger set of information, the classification errors decreased significantly. The best combination of five metabolites was found to have misclassification errors as low as 3%. This combination included one metabolite from the FOCM/TS metabolites (Glu-Cys). At least for this study, the metabolites of the FOCM/TS pathway provide some information for a modest classification, but other metabolites play an even more important role. Correlation analysis (Table 8) revealed that there appear to be five primary categories of significantly different metabolites, with significant correlations within the group to the primary metabolites, but low correlations between the 5 primary metabolites. Almost all the metabolites which were significantly different between the ASD-M and TD-M groups (see Tables 5) fell into one of these five groups. However, there were five metabolites that did not significantly correlate with any of the primary metabolites and did not correlate with each other.

### Carnitine-conjugated metabolites

The univariate analysis found that all but one carnitine-conjugated metabolite (Adrenoylcarnitine (C22:4)*) were significantly lower in the ASD-M group, with the ratio of carnitine levels for ASD-M/TD-M ranging from 0.66 to 0.87, with an average of 0.78. Carnitine can be produced by the body, but there is some dietary intake also, with the only common dietary sources of carnitine being beef and (to a lesser extent) pork. There were no significant differences in the beef consumption quantity and frequency between the two groups of mothers. Similarly, the levels of carnitine and two of its precursors (lysine and trimethyllysine) were essentially identical for the ASD-M and TD-M groups (data not shown). This suggests a difference in the process of carnitine conjugation and may be due to a defect or impairment of the enzymes which control carnitine conjugation, such as carnitine palmitoyltransferase 1 (CPT1). Other studies have also shown that carnitine supplementation is beneficial for children with ASD [35–37].

### Implication on possible role of nutritional/metabolic status of mothers with children with ASD

Couples who have had a child with ASD have an 18.7% chance of future children being diagnosed with ASD [16], while the general risk for ASD is approximately 1.9% [15]. While it is not known if a mother’s metabolic profile is linked to their child’s ASD diagnosis, this is a hypothesis that requires further research. Our results indicate that measurements of Glu-Cys, histidylglutamate, cinnamoylglycine, proline, and adrenoylcarnitine (C22:4)* may be able to predict with approximately 97% accuracy whether a woman, while she is not pregnant, had a child with ASD in the previous 2–5 years. It should be noted that it is not possible from this study to draw conclusions about nutritional status of the mothers during pregnancy.

This research found that several metabolites of the FOCM/TS pathway are different in the ASD-M group. One previous study found that several metabolites from the FOCM/TS pathway were significantly different in children with ASD as compared to controls [2]. A second study measured these metabolites in non-pregnant mothers, similar to what was done here, and found using univariate analysis that homocysteine, adenosine, and SAH were elevated in the mothers who have a child with ASD [7], but the work did not perform the classification analysis that this paper covers. These previous studies, in combination with the research done here, serve as indicators that the FOCM/TS pathways are of importance to ASD research. That being said, this work also tried to replicate findings from a previous study involving two groups of pregnant mothers (mothers who have had a child with ASD and mothers who have not) using metabolites from the FOCM/TS pathways [8]. However, it was found that the FOCM/TS metabolites alone provided only modest classification ability. It needs to be emphasized that this study focuses on metabolic measurements 2–5 years after birth while the significantly stronger results from [8] involved measurements during pregnancy. As such the two results are not directly comparable.

Several studies have suggested that other pathways may be indicative of ASD risk. Studies have found abnormalities in glutathione metabolism and redox metabolism in people with ASD. These processes are important for cell health and can affect other processes within the FOCM/TS pathways [38]. Another study found that when a mother had diabetes and was obese pre-pregnancy, her branched-chain amino acid level was significantly associated with the risk of her child having autism [39]. Similar to that study, further research has found that combining low maternal high-density lipoprotein cholesterol along with high maternal plasma branched-chain amino acid levels and the child being male led to an increased risk of the child being diagnosed with ASD [40]. Another study involving maternal metabolite levels pre-pregnancy from blood samples stored from that time, found that mothers who had children with ASD showed differences in several metabolic pathways including bile acid pathways, glycosphingolipid synthesis, *N*-glycan and pyrimidine metabolism, and C21-steroid hormone biosynthesis and metabolism [41].

It is important to note that the results for this pilot study are for maternal levels post-pregnancy, so they are only suggestive of possible nutritional and metabolic differences during pregnancy.

### Limitations of this study

There were several limitations of the study performed. This is a pilot study with a relatively small sample size. Further studies with larger sample sizes are needed to validate these results and to confirm that the medical histories, symptoms, and current medications of the mothers are not cofounders for the metabolic analysis. These further studies would also benefit by including these potential cofounders in the multivariate analysis. Further studies should also consider measuring paternal factors such as age and diet as a potential confounder. Metabolite concentrations do not stay constant as stress and diet can have an effect. Some of these issues can be a factor of when they were measured. Also, the measurements were taken 2–5 years after giving birth and therefore, the measurements are only suggestive of differences that might have been present during pregnancy/lactation. There was also a discrepancy in the ages of the children in this study. The ages of the mothers were closely matched, but the time since giving birth is different as the children in the ASD group were slightly older (4.71 +/− 1.0 vs. 3.87 +/− 1.3, *p* = 0.01). Future studies should try to match ages of the mother and time since birth. This is especially important as the diet information was collected using a block food frequency questionnaire for when the mothers were pregnant which may result in a recall bias the more time had passed since birth. Another potential limitation of using this questionnaire is a response bias where some of the dietary intake levels may be underestimated due to social expectations.

## Conclusions

In conclusion, this study found many statistically significant differences in metabolites of mothers of children with ASD compared to mothers of typically-developing children, at 2–5 years after birth. A subset of five metabolites was sufficient to differentiate the two groups with approximately 97% accuracy, after leave-one-out cross-validation. Almost all of the metabolites that were significantly different between the two groups were correlated with one of these five metabolites, suggesting that there are at least five areas of metabolic differences between the ASD-M group and the TD-M groups, represented by the core metabolites (Glu-Cys, histidylglutamate, cinnamoylglycine, proline, adrenoylcarnitine (C22:4)) which each correlated with many others. The results of this pilot study may be useful for guiding future studies of metabolic risk factors during conception/pregnancy/lactation.

## Supplementary Information

**Additional file 1: Table S-1.** Contains a summary of self-reported data from the mothers about their children. The child’s developmental history was asked in the form of a multiple-choice question of the ASD-M group of which category of development the child belonged in. These categories are listed in Table S-1.

**Additional file 2: Table S-2.** The mothers in this study answered questions about characteristics and conditions during their pregnancy and that information is shown in Table S-2.

**Additional file 3: Table S-3**. The full Metabolon dataset contained 595 metabolite measurements. The 50 metabolites with the highest AUC, shown in Table 5, were included in the multivariate analysis. 45 of these metabolites exhibited statistically significant differences in the mean or median between the two groups. There were three other metabolites in the full Metabolon dataset that were not included in the multivariate analysis because they had lower AUC values than those in the top 50. These metabolites and their hypothesis testing results are shown in Table S-3.

**Additional file 4: Table S-4.** Lists the pathways and subpathways of the 50 metabolites that were measured by Metabolon and included in the analysis.

**Additional file 5: Table S-5.** The best combination of metabolites was reported in Table 7. There were combinations involving fewer metabolites that produced reasonably low misclassification errors. These combinations and their errors are reported in Table S-5.

**Additional file 6: Table S-6.** There were other combinations of five metabolites of subset (iv) that produced similar type I/type II errors as the best combination reported in Table 7. These combinations and their errors are shown in Table S-6.

**Additional File 7: Raw Data File.** The excel file contains the raw data of the metabolite measurements analyzed in this paper. The metabolite measurements are separated by tabs by what lab measured them. The three labs included the Metabolic and Oxidative Stress Laboratory (MOSL), the Mayo Clinic, and Metabolon.

## Data Availability

The datasets supporting the conclusions of this article are included within the article’s additional files.

## Abbreviations

ASD: Autism Spectrum Disorder
TD: Typically-Developing
FOCM: Folate One-Carbon Metabolism
TS: Transsulfuration
ASD-M: Mothers of young children with Autism Spectrum Disorder
TD-M: Mothers of typically-developing young children
AMPSYCAM: ASU-Mayo Pilot Study of Young Children with ASD and their Mothers
ADI-R: Autism Diagnostic Interview-Revised
MMA: Methylmalonic acid
LC-MS/MS: Liquid Chromatography Tandem Mass Spectrometry
MOSL: The Metabolic and Oxidative Stress Laboratory
HPLC: High-performance liquid chromatography
UHPLC/MS: Ultra high-performance liquid chromatography
LC/MS Pos Polar: Reverse phase chromatography positive ionization method optimized for hydrophilic compounds
LC/MS Pos Lipid: Reverse phase chromatography positive ionization method optimized for hydrophobic compounds
LC/MS Neg: Reverse phase chromatography with negative ionization conditions
HILIC: Hydrophilic interaction liquid chromatography
LC/MS Polar: HILIC method coupled to negative
t =: Student’s t-test
t ≠: Welch t-test
MW: Mann-Whitney U test
t ≠ †: Welch t-test without normality criteria being met
*χ*^2^: Chi-squared test
FDR: False discovery rate
AUC: Area under the curve
ROC: Receiver operating characteristic
FPR: False positive rate
TPR: True positive rate
FDA: Fisher Discriminant Analysis
SAM: S-adenosylmethionine
SAH: S-adenosylhomocysteine
GSH: Glutathione
GSSG: Oxidized glutathione
LOOCV: Leave-one-out cross-validation
GI: Gastrointestinal
PDF: Probably density function
CPT1: Carnitine palmitoyltransferase 1
