# Supplementary material for: Altered metabolism of mothers of young children with Autism Spectrum Disorder: a case control study
Source: BMC Pediatr. 2020 Dec 14;20:557. doi: 10.1186/s12887-020-02437-7 (PMC7734806; doi:10.1186/s12887-020-02437-7)
Supplement: Supplementary file 1 — Additional file 1: Table S-1. Contains a summary of self-reported data from the mothers about their children. The child’s developmental history was asked in the form of a multiple-choice question of the ASD-M group of which category of development the child belonged in. These categories are listed in Table S-1. [file 12887_2020_2437_MOESM1_ESM.docx]

**Table S-1**

*Medical histories and characteristics of children.*

|  | **ASD (n=30)** | **TD (n=29)** | **p-Value of t-test (T) or Chi-Squared (C)** | **FDR** |
| --- | --- | --- | --- | --- |
| **Child sex** | 22 m, 8 f (73% male) | 14 m, 14 f (50% male) | 0.05 (C) | n.s. |
| **Child age (years)** | 4.71 (1.0) | 3.87 (1.3) | 0.0091 (T) | 0.00 |
| **Child birthweight (lbs.)** | 7.21 (4.2) | 6.20 (4.7) | n.s. (T) |  |
| ***Child’s Antibiotic usage (Rounds, where 1 round=10 days)*** | | | | |
| **0-6 months** | 0.22 (0.6) | 0.29 (0.5) | n.s. (T) |  |
| **6-12 months** | 0.73 (1.3) | 0.79 (1.0) | n.s. (T) |  |
| **12-24 months** | 1.08 (2.0) | 1.25 (1.7) | n.s. (T) |  |
| **24-36 months** | 1.12 (1.1) | 0.74 (1.1) | n.s. (T) |  |
| **36-48 months** | 0.77 (1.1) | 0.36 (0.6) | n.s. (T) |  |
| **Total antibiotic usage 0-48 months** | 3.29 (4.1) | 3.24 (3.0) | n.s. (T) |  |
| **Child’s Asthma severity** | 3%  1 mild | 18%  3 mild, 1 moderate, 1 severe | n.s. (C) |  |
| **Child’s Food allergies/sensitivities (severity based on most severe allergy)** | 32%  (7% mild  14% moderate  11% severe) | 11%  (4% mild, 7% moderate  0% severe) | n.s. (C) |  |
| **Other allergies**  **(severity based on most severe allergy)** | 14%  (7% mild, 4% moderate, 4% unknown) | 33%  (19% mild, 15% moderate, 0% severe) | n.s. (C) |  |
| ***Child’s Developmental history of ASD*** | | | | |
| **Early Onset** | 14% |  |  |  |
| **Normal development, then regression (age of regression)** | 46%  19 (5) months |  |  |  |
| **Normal development, then plateau (age of plateau)** | 39%  19 (9) months |  |  |  |
