# Supplementary material for: Altered metabolism of mothers of young children with Autism Spectrum Disorder: a case control study
Source: BMC Pediatr. 2020 Dec 14;20:557. doi: 10.1186/s12887-020-02437-7 (PMC7734806; doi:10.1186/s12887-020-02437-7)
Supplement: Supplementary file 2 — Additional file 2: Table S-2. The mothers in this study answered questions about characteristics and conditions during their pregnancy and that information is shown in Table S-2. [file 12887_2020_2437_MOESM2_ESM.docx]

**Table S-2**

*Characteristics, dietary, and medical history of pregnancy of mothers.*

|  | **ASD (n=30)** | **TD (n=29)** | **p-Value of t-test (T) or Chi-Squared (C)** | **FDR** |
| --- | --- | --- | --- | --- |
| **Pregnancy complications** | 43%  (18% mild, 18% moderate, 7% severe | 39%  (25% mild, 14% moderate, 0% severe) | n.s. (C) |  |
| **Birth complications** | 50%  (36% mild, 11% moderate, 4% severe) | 32%  (21% mild, 7% moderate, 4% severe) | n.s. (C) |  |
| **C-section** | 43% | 29% | n.s. (C) |  |
| **Months of Breastfeeding without formula** | 9.1 (11) | 8.4 (9.6) | n.s. (T) |  |
| **Months of Breastfeeding with formula** | 2.9 (4.8) | 4.3 (7.1) | n.s. (T) |  |
| **Months of formula only** | 4.4 (5.7) | 3.1 (4.2) | n.s. (T) |  |
| **Solids introduced** | 6.4 (1.3) | 6.2 (2.6) | n.s. (T) |  |
| **Prenatal usage** | 89% | 93% | n.s. (C) |  |
| **% used prenatal prior to conception** | 37% | 37% |  |  |
| **Week started prenatal (with preconception use scored as week zero)** | 2.8 (3) | 3.8 (4) | n.s. (T) |  |
| **Pesticide exposure** | 14% | 11% | n.s. (C) |  |
| **% organic food** | 24% (26%) | 24% (25%) | n.s. (T) |  |

*Note. The prenatal usage is measured as the percentage of mothers who used any prenatal supplements. This is the same for the pesticide exposure – the percentage of mothers who were exposed to any pesticides. Organic foods refers to the USDA definition of organic foods that are free from pesticides.*
