# Supplementary material for: Altered metabolism of mothers of young children with Autism Spectrum Disorder: a case control study
Source: BMC Pediatr. 2020 Dec 14;20:557. doi: 10.1186/s12887-020-02437-7 (PMC7734806; doi:10.1186/s12887-020-02437-7)
Supplement: Supplementary file 3 — Additional file 3: Table S-3. The full Metabolon dataset contained 595 metabolite measurements. The 50 metabolites with the highest AUC, shown in Table 5, were included in the multivariate analysis. 45 of these metabolites exhibited statistically significant differences in the mean or median between the two groups. There were three other metabolites in the full Metabolon dataset that were not included in the multivariate analysis because they had lower AUC values than those in the top 50. These metabolites and their hypothesis testing results are shown in Table S-3. [file 12887_2020_2437_MOESM3_ESM.docx]

Table S-3

Metabolites from the Metabolon dataset not included in the top 50 for analysis with significant p-values and FDR values.

| **Metabolite** | **Test** | **p-Value** | **FDR** | **AUC** |
| --- | --- | --- | --- | --- |
| 2-hydroxyphenylacetate | t= | 0.02 | 0.02 | 0.66 |
| N-acetylleucine | MW | 0.02 | 0.02 | 0.66 |
| Margaroylcarnitine (C17)* | t$\neq$ | 0.02 | 0.04 | 0.65 |

*Note. The p-Values, FDR values, and AUC values are listed in the table and sorted by AUC value (largest to smallest), p-value (smallest to largest), and then FDR value (smallest to largest). The hypothesis tests are Student’s t-test (t=), Mann-Whitney U test (MW), and Welch’s test (t*$\neq$*). The * indicates metabolites measured by Metabolon that have not been officially confirmed based on a standard, but Metabolon is confident in the metabolite’s identity.*
