# Supplementary material for: Altered metabolism of mothers of young children with Autism Spectrum Disorder: a case control study
Source: BMC Pediatr. 2020 Dec 14;20:557. doi: 10.1186/s12887-020-02437-7 (PMC7734806; doi:10.1186/s12887-020-02437-7)
Supplement: Supplementary file 4 — Additional file 4: Table S-4. Lists the pathways and subpathways of the 50 metabolites that were measured by Metabolon and included in the analysis. [file 12887_2020_2437_MOESM4_ESM.docx]

Table S-4

Pathways and subpathways of the metabolites from the broad metabolomics.

| **Metabolite** | **Pathway** | **Sub-Pathway** | **Higher/lower in ASD-M group** |
| --- | --- | --- | --- |
| N-acetylasparagine $\Delta$ | Amino Acid | Alanine and Aspartate Metabolism | ↓ |
| S-1-pyrroline-5-carboxylate $\Delta$ | Amino Acid | Glutamate Metabolism | ↓ |
| Glutamine $\Delta$ | Amino Acid | Glutamate Metabolism | ↓ |
| Alpha-ketoglutaramate* $\Delta$ | Amino Acid | Glutamate Metabolism | ↓ |
| 5-oxoproline $\Delta$ | Amino Acid | Glutathione Metabolism | ↓ |
| S-methylglutathione $\Delta$ | Amino Acid | Glutathione Metabolism | ↓ |
| Glycine | Amino Acid | Glycine, Serine and Threonine Metabolism | ↓ |
| Isovalerylglycine $\Delta$ | Amino Acid | Leucine, Isoleucine and Valine Metabolism | ↓ |
| N-acetylvaline $\Delta$ | Amino Acid | Leucine, Isoleucine and Valine Metabolism | ↓ |
| Tiglylcarnitine (C5:1-DC) $\Delta$# | Amino Acid | Leucine, Isoleucine and Valine Metabolism | ↓ |
| 5-methylthioadenosine (MTA) $\Delta$ | Amino Acid | Polyamine Metabolism | ↓ |
| N-formylanthranilic acid $\Delta$ | Amino Acid | Tryptophan Metabolism | ↓ |
| 3-indoxyl sulfate $\Delta$ | Amino Acid | Tryptophan Metabolism | ↓ |
| Phenol sulfate $\Delta$ | Amino Acid | Tyrosine Metabolism | ↓ |
| Citrulline $\Delta$ | Amino Acid | Urea cycle; Arginine and Proline Metabolism | ↓ |
| Proline $\Delta$ | Amino Acid | Urea cycle; Arginine Proline Metabolism | ↓ |
| Mannose | Carbohydrate | Fructose, Mannose and Galactose Metabolism | ↑ |
| Fructose $\Delta$ | Carbohydrate | Fructose, Mannose, and Galactose Metabolism | ↓ |
| Nicotinamide adenine dinucleotide (NAD+) $\Delta$ | Cofactors and Vitamins | Nicotinate and Nicotinamide Metabolism | ↓ |
| Pyridoxate $\Delta$ | Cofactors and Vitamins | Vitamin B6 Metabolism | ↓ |
| Succinylcarnitine (C4-DC) $\Delta$# | Energy | TCA Cycle | ↓ |
| N-palmitoylserine $\Delta$ | Lipid | Endocannabinoid | ↓ |
| Decanoylcarnitine (C10) $\Delta$# | Lipid | Fatty Acid Metabolism (Acyl Carnitine) | ↓ |
| Octanoylcarnitine (C8) $\Delta$# | Lipid | Fatty Acid Metabolism (Acyl Carnitine) | ↓ |
| Cis-4-decenoylcarnitine (C10:1) $\Delta$# | Lipid | Fatty Acid Metabolism (Acyl Carnitine) | ↓ |
| Arachidoylcarnitine (C20)* $\Delta$# | Lipid | Fatty Acid Metabolism (Acyl Carnitine) | ↓ |
| Myristoylcarnitine (C14) $\Delta$# | Lipid | Fatty Acid Metabolism (Acyl Carnitine) | ↓ |
| Laurylcarnitine (C12) $\Delta$# | Lipid | Fatty Acid Metabolism (Acyl Carnitine) | ↓ |
| Stearoylcarnitine (C18) $\Delta$# | Lipid | Fatty Acid Metabolism (Acyl Carnitine) | ↓ |
| Docosapentaenoylcarnitine (C22:5n3)* $\Delta$# | Lipid | Fatty Acid Metabolism (Acyl Carnitine) | ↓ |
| Arachidonoylcarnitine (C20:4) $\Delta$# | Lipid | Fatty Acid Metabolism (Acyl Carnitine) | ↓ |
| Lignoceroylcarnitine (C24)* $\Delta$# | Lipid | Fatty Acid Metabolism (Acyl Carnitine) | ↓ |
| Palmitoylcarnitine (C16) $\Delta$# | Lipid | Fatty Acid Metabolism (Acyl Carnitine) | ↓ |
| Eicosenoylcarnitine (C20:1)* $\Delta$# | Lipid | Fatty Acid Metabolism (Acyl Carnitine) | ↓ |
| Adrenoylcarnitine (C22:4)*# | Lipid | Fatty Acid Metabolism (Acyl Carnitine) | ↓ |
| N-palmitoylglycine $\Delta$ | Lipid | Fatty Acid Metabolism (Acyl Glycine) | ↓ |
| Propionylglycine $\Delta$ | Lipid | Fatty Acid Metabolism (also BCAA Metabolism) | ↓ |
| N-acetyl-2-aminooctanoate*$\Delta$ | Lipid | Fatty Acid, Amino | ↓ |
| Histidylglutamate $\Delta$ | Peptide | Dipeptide | ↑ |
| Asparaginylalanine $\Delta$ | Peptide | Dipeptide | ↑ |
| Gamma-glutamylglycine $\Delta$ | Peptide | Gamma-glutamyl Amino Acid | ↓ |
| Gamma-glutamyltyrosine $\Delta$ | Peptide | Gamma-glutamyl Amino Acid | ↓ |
| 4-vinylphenol sulfate $\Delta$ | Xenobiotics | Benzoate Metabolism | ↓ |
| Catechol sulfate $\Delta$ | Xenobiotics | Benzoate Metabolism | ↓ |
| Guaiacol sulfate $\Delta$ | Xenobiotics | Benzoate Metabolism | ↓ |
| 6-hydroxyindole sulfate $\Delta$ | Xenobiotics | Chemical | ↓ |
| Dimethyl sulfone | Xenobiotics | Chemical | ↑ |
| Cinnamoylglycine $\Delta$ | Xenobiotics | Food Component/Plant | ↓ |
| 7-methylxanthine $\Delta$ | Xenobiotics | Xanthine Metabolism | ↓ |
| 3-methylxanthine | Xenobiotics | Xanthine Metabolism | ↓ |

*Note. The metabolites listed here are the 50 metabolites measured by Metabolon from broad metabolomics with the highest area under the receiver operating characteristic (ROC) curve (AUC). The metabolites are sorted alphabetically by pathway and then subpathway. A fourth column lists whether the metabolites were higher or lower in the ASD-M group. Metabolites that had a p-value ≤0.05 and FDR ≤ 0.1 (FDR-values listed in Table 3) are marked by* $\Delta$*. The * indicates a metabolite that has not been officially confirmed based on a standard, but Metabolon is confident in its identity. The # indicates carnitine-conjugated metabolites.*
