# Supplementary material for: Altered metabolism of mothers of young children with Autism Spectrum Disorder: a case control study
Source: BMC Pediatr. 2020 Dec 14;20:557. doi: 10.1186/s12887-020-02437-7 (PMC7734806; doi:10.1186/s12887-020-02437-7)
Supplement: Supplementary file 5 — Additional file 5: Table S-5. The best combination of metabolites was reported in Table 7. There were combinations involving fewer metabolites that produced reasonably low misclassification errors. These combinations and their errors are reported in Table S-5. [file 12887_2020_2437_MOESM5_ESM.docx]

Table S-5

Multivariate results using top combinations of 2-4 variables from subset (iv).

| **Metabolites** | **Type I Error (FPR)** | **Type II Error (FNR)** |
| --- | --- | --- |
| 2 metabolites:  Histidylglutamate, 6-hydroxyindole sulfate | 17% | 13% |
| 3 metabolites:  Histidylglutamate, N-formylanthranilic acid, palmitoylcarnitine (C16) | 7% | 7% |
| 4 metabolites: Histidylglutamate, S-1-pyrroline-5-carboxylate, N-acetyl-2-aminooctanoate*, 5-methylthioadenosine (MTA) | 3% | 7% |

*Note. The * indicates metabolites measured by Metabolon that were not officially confirmed based on a standard, but Metabolon is confident of the Metabolite’s identity.*
