# Supplementary material for: Altered metabolism of mothers of young children with Autism Spectrum Disorder: a case control study
Source: BMC Pediatr. 2020 Dec 14;20:557. doi: 10.1186/s12887-020-02437-7 (PMC7734806; doi:10.1186/s12887-020-02437-7)
Supplement: Supplementary file 6 — Additional file 6: Table S-6. There were other combinations of five metabolites of subset (iv) that produced similar type I/type II errors as the best combination reported in Table 7. These combinations and their errors are shown in Table S-6. [file 12887_2020_2437_MOESM6_ESM.docx]

Table S-6

Multivariate results using the top combinations of five variables from subset (iv).

| **Metabolites** | **Type I Error (FPR)** | **Type II Error (FNR)** |
| --- | --- | --- |
| SAM/SAH, percent oxidized, histidylglutamate, cis-4-decenoylcarnitine (C10:1), 3-indoxyl sulfate | 3% | 7% |
| fGSH/GSSG, histidylglutamate, 4-vinylphenol sulfate, 3-indroxyl sulfate, palmitoylcarnitine (C16) | 3% | 7% |
| Histidylglutamate, 4-vinylphenol sulfate, cinnamoylglycine, N-acetylvaline, palmitoylcarnitine (C16) | 3% | 7% |
| Glu-Cys, histidylglutamate, catechol sulfate, phenol sulfate, N-acetyl-2-aminooctanoate* | 3% | 7% |
| tGSH, 4-vinylphenol sulfate, 5-oxoproline, asparaginylalanine, tiglylcarnitine (C5:1-DC) | 7% | 3% |

*Note. The * indicates a metabolite measured by Metabolon that has not been officially confirmed based on a standard, but Metabolon is confident in the identity.*
